# Supplementary material for: Dimension matters when modeling network communities in hyperbolic spaces
Source: PNAS Nexus. 2023 Apr 18;2(5):pgad136. doi: 10.1093/pnasnexus/pgad136 (PMC10167553; doi:10.1093/pnasnexus/pgad136)
Supplement: pgad136_Supplementary_Data [file pgad136_supplementary_data.zip › PNASNEXUS-PNASNEXUS-2022-01001-s02.pdf]

Supporting Information for

Dimension matters when modeling network communities in hyperbolic spaces

Béatrice Désy, Patrick Desrosiers and Antoine Allard

Antoine Allard.
E-mail: antoine.allard@phy.ulaval.ca

This PDF file includes:

Supporting text
Fig. S1
SI References

Supporting Information Text

1. Explicit computations for angular densities

A. Modes of angular distance between connected node pairs. Taking the first derivative of Eq. (17) with respect to θ and setting it to zero yields

$$\frac{(D-1)\sin^{D-2}\theta^*\cos\theta^*}{1+(\theta^*/\eta)^\beta} = \frac{\sin^{D-1}\theta^*}{[1+(\theta^*/\eta)^\beta]^2}\frac{\beta(\theta^*/\eta)^\beta}{\theta^*}, \tag{S1}$$

where θ^* can be a minimum, a maximum or an inflexion point. We separate two cases:

1. If $\theta^* \in (0, \pi)$, Eq. (S1) can be simplified to Eq. (19).
2. We proceed to show that $\theta^* = 0$ iff $D = 1$ in the regime $\beta > D$, $\eta > 0$. First, if $D = 1$, Eq. (S1) reduces to

   $$0 = \frac{\beta(\theta^*/\eta)^\beta}{\theta^*[1+(\theta^*/\eta)^\beta]^2}, \tag{S2}$$

   which is verified only for $\theta^* = 0$. Conversely, setting $\theta^* = 0$ in Eq. (S1) trivially yields $D = 1$.

B. Probability density function for η. Since η is a scalar function of κ, κ', the pdf for Y can be computed as follows.

$$f_Y(\eta) = \int_{\Omega_K}\int_{\Omega_K} \rho_K(\kappa)\rho_K(\kappa')\delta(g(\kappa'))\,\mathrm{d}\kappa\,\mathrm{d}\kappa' \tag{S3}$$

with $g(\kappa') := \eta - (\mu\kappa\kappa')^{1/D}/R$ and δ the Dirac delta function. By definition of composition with the Dirac delta function under the integral,

$$\delta(g(\kappa')) = \frac{\delta(\kappa'-\kappa'_*)}{|g'(\kappa'_*)|}, \tag{S4}$$

with $\kappa'_* = (\eta R)^D/\mu\kappa$, the unique root of g. Hence

$$\left.\frac{\mathrm{d}g}{\mathrm{d}\kappa'}\right|_{\kappa'=\kappa'_*} = \frac{\mu\kappa\eta^{(1-D)}}{DR^D} > 0, \tag{S5}$$

and it follows that

$$\delta(g(\kappa')) = \frac{\delta(\kappa' - (\eta R)^D/\mu\kappa)}{\mu\kappa\eta^{(1-D)}}DR^D. \tag{S6}$$

Eq. (S3) can thus be computed as

$$f_Y(\eta) = \frac{DR^D}{\mu\eta^{1-D}}\int_0^\infty \rho_\kappa(\kappa)\rho_\kappa((\eta R)^D/\mu\kappa)\frac{d\kappa}{\kappa}. \tag{S7}$$

For any distribution of κ with a non-zero lower bound κ_0,

$$\frac{(\eta R)^D}{\mu\kappa} < \kappa_0 \quad\Longleftrightarrow\quad \frac{(\eta R)^D}{\mu\kappa_0} < \kappa, \tag{S8}$$

which means that the integrand in Eq. (S7) is null in the regime of Eq. (S8). We finally have

$$f_Y(\eta) = \frac{DR^D}{\mu\eta^{1-D}}\int_{\kappa_0}^{(\eta R)^D/\mu\kappa_0} \rho_\kappa(\kappa)\rho_\kappa((\eta R)^D/\mu\kappa)\frac{d\kappa}{\kappa}. \tag{S9}$$

This pdf can be computed exactly for κ drawn from a Pareto distribution with parameter γ. Let

$$\rho_\kappa(\kappa) = (\gamma-1)\kappa_0^{\gamma-1}\kappa^{-\gamma}, \tag{S10}$$

then computation of the integral in Eq. (S9) gives

$$f_Y^{\text{Pareto}}(\eta) = \frac{D(\gamma-1)^2\kappa_0^{2(\gamma-1)}\mu^{\gamma-1}}{R^{D(\gamma-1)}\eta^{D(\gamma-1)+1}}\log\left[\frac{(\eta R)^D}{\mu\kappa_0^2}\right]. \tag{S11}$$

C. Marginalized connection probability. Since the connection probability of hyperbolic random graphs (Eq. (5)) depends on θ and η, one would need to know the pdf for θ and the pdf for η to compute the marginalized connection probability that appears in the normalization of Eq. (24).

$$f_A(1) = \int_{\Omega_Y} \int_{\Omega_X} f_{X,Y,A}(\theta, \eta, 1)\, \mathrm{d}\theta\, \mathrm{d}\eta. \tag{S12}$$

Given $\Omega_X = [0, \pi]$, $\Omega_Y = [\eta_0, \infty)$ and Eq. (14),

$$f_A(1) = \int_{\eta_0}^{\infty} \int_0^{\pi} \frac{\sin^{D-1}\theta\, f_Y(\eta)}{I_D[1 + (\theta/\eta)^\beta]}\, \mathrm{d}\theta\, \mathrm{d}\eta \tag{S13}$$

$$= \frac{1}{I_D} \int_{\eta_0}^{\infty} \left[\int_0^{\pi} \frac{\sin^{D-1}\theta}{1 + (\theta/\eta)^\beta}\, \mathrm{d}\theta \right] f_Y(\eta)\, \mathrm{d}\eta \tag{S14}$$

$$= \frac{\sqrt{\pi}\,\Gamma(D/2)}{\Gamma(D+1/2)} \int_{\eta_0}^{\infty} \mathcal{Z}(\eta) f_Y(\eta)\, \mathrm{d}\eta, \tag{S15}$$

where the angular distance distribution of Eq. (11) and the definition of Eq. (18) have been used.

2. Sampling methods

To sample clusters of angular coordinates for hyperbolic random graphs in $D = 2$, we first distribute the modes of all n clusters evenly using the Fibonacci lattice algorithm [1]. Then, coordinates of nodes within each cluster are sampled from a three-dimensional normal distribution in $\mathbb{R}^3$ centered around its mode, and then projected on the unit sphere. Lastly, the community to which each node belongs is defined as the one of the closest mode, or as the closest centroid. This allows us to obtain a given number n of evenly distributed clusters of angular coordinates for the nodes.

3. Other thresholding methods for block matrices

Here we validate that results about community degree of Fig. 9 are robust to other binarization methods. In Fig. S1, we show the community degree $\langle k \rangle$ measured on the same matrices as the ones used for Fig. 9, but using a higher threshold (left) and the disparity filter of Ref. [2] (right) to transform the inter-community edges probability matrix B to a binary matrix. Both plots show that community degree in $\mathbb{S}^2$ increases to higher values than in $\mathbb{S}^1$. The disparity filter penalizes locally homogeneous edge weights, which explains why the blue curves on the right panel do not increase as much as in Fig. 9, since then the inter-community edge probability becomes more and more homogeneous on the neighboring angular clusters.

References

1. A González, Measurement of Areas on a Sphere Using Fibonacci and Latitude–Longitude Lattices. *Math. Geosci.* **42**, 49 (2009).
2. MÁ Serrano, M Boguñá, A Vespignani, Extracting the multiscale backbone of complex weighted networks. *Proc. Natl. Acad. Sci.* **106**, 6483–6488 (2009).

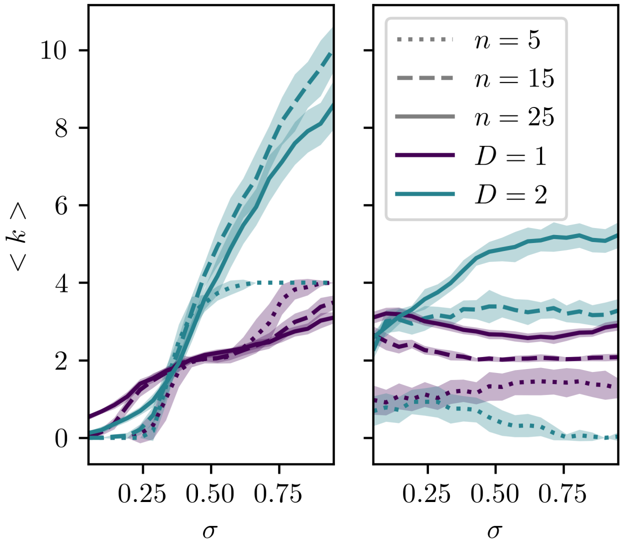

Fig. S1. Community degree $\langle k \rangle$ with alternative binarization methods. On the left, Eq. (37) is used with a higher threshold of $10/m$ and on the right, the disparity filter of Ref. [2] is applied with $\alpha = 0.2$.
