# Supplementary figures and images for: Dimension matters when modeling network communities in hyperbolic spaces
Source: PNAS Nexus. 2023 Apr 18;2(5):pgad136. doi: 10.1093/pnasnexus/pgad136 (PMC10167553; doi:10.1093/pnasnexus/pgad136)

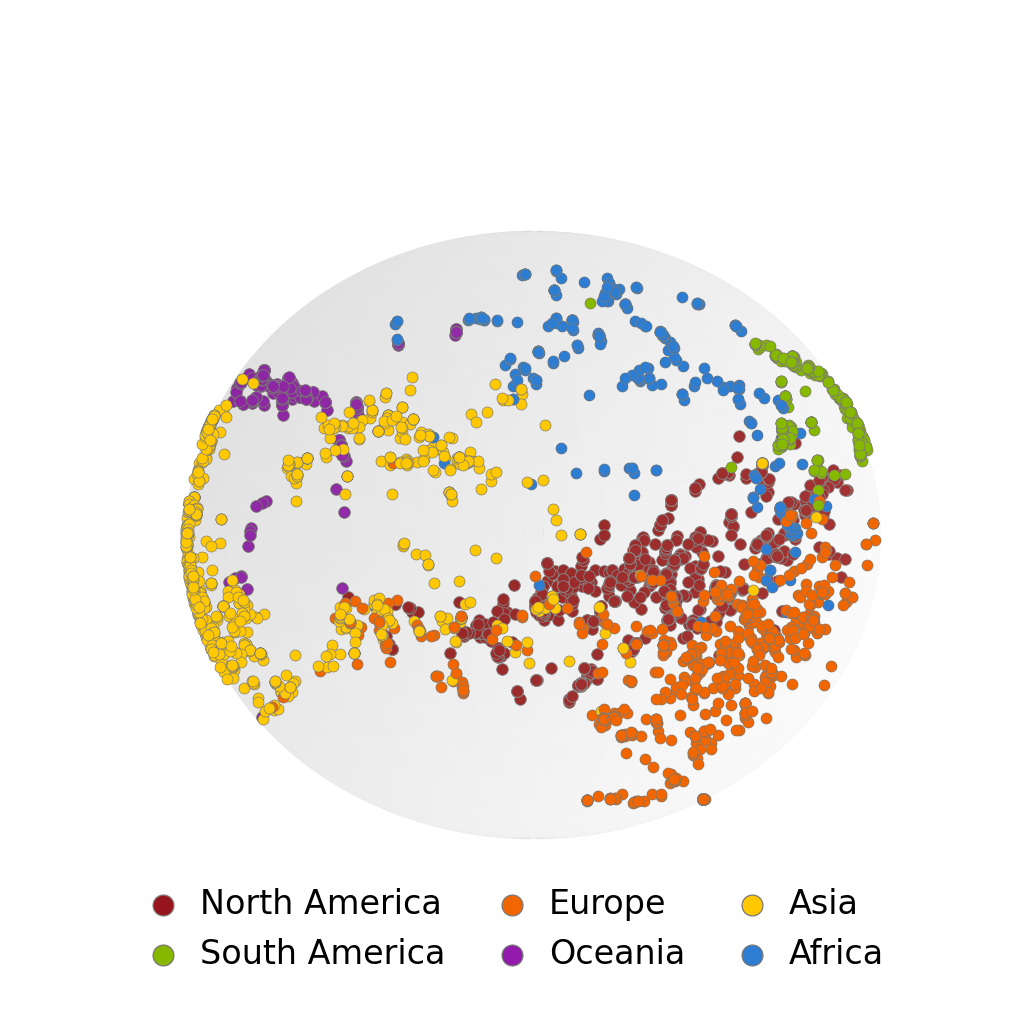

Supplement: pgad136_Supplementary_Data [file pgad136_supplementary_data.zip › PNASNEXUS-PNASNEXUS-2022-01001-s01.gif]
